# Stepwise neuronal network pattern formation in agarose gel during cultivation using non-destructive microneedle photothermal microfabrication

**DOI:** 10.1038/s41598-021-93988-x

**Published:** 2021-07-19

**Authors:** Yuhei Tanaka, Haruki Watanabe, Kenji Shimoda, Kazufumi Sakamoto, Yoshitsune Hondo, Mitsuru Sentoku, Rikuto Sekine, Takahito Kikuchi, Kenji Yasuda

**Affiliations:** 1 Department of Pure and Applied Physics, Graduate School of Advanced Science and Engineering, Waseda University, Tokyo, 169-8555 Japan; 2 Department of Physics, School of Advanced Science and Engineering, Waseda University, Tokyo, 169-8555 Japan

**Keywords:** Lab-on-a-chip, Neural circuits, Cellular neuroscience, Tissue engineering, Biomaterials - cells, Assay systems, Dynamic networks

## Abstract

Conventional neuronal network pattern formation techniques cannot control the arrangement of axons and dendrites because network structures must be fixed before neurite differentiation. To overcome this limitation, we developed a non-destructive stepwise microfabrication technique that can be used to alter microchannels within agarose to guide neurites during elongation. Micropatterns were formed in thin agarose layer coating of a cultivation dish using the tip of a 0.7 $\upmu \mathrm{m}$-diameter platinum-coated glass microneedle heated by a focused 1064-nm wavelength infrared laser, which has no absorbance of water. As the size of the heat source was 0.7 $\upmu \mathrm{m}$, which is smaller than the laser wavelength, the temperature fell to 45 $^\circ \hbox {C}$ within a distance of 7.0 $\upmu \mathrm{m}$ from the edge of the etched agarose microchannel. We exploited the fast temperature decay property to guide cell-to-cell connection during neuronal network cultivation. The first neurite of a hippocampal cell from a microchamber was guided to a microchannel leading to the target neuron with stepwise etching of the micrometer resolution microchannel in the agarose layer, and the elongated neurites were not damaged by the heat of etching. The results indicate the potential of this new technique for fully direction-controlled on-chip neuronal network studies.

## Introduction

Microfabrication technologies have enabled us to study the constructive approach of cell-to-cell interactions and single cell-based cellular network analysis within the confined structures. Especially as the spatial neuronal connection patterns are implicated to play a crucial role in the perception and processing of signals as the information, simple cell culture-based neuronal networks were examined. And researches have shown that the formed chemical and electrical synapses in cultured neuronal networks exhibited short-term modulations similar to those in brain slices^1^.

Several microfabrication technologies, such as microprinting and microstructural design, can be used to form neuronal networks in geometric patterns. In general, micropatterning technologies are simple but powerful to allow precise control of spatial neuron-to-neuron connections^1–8^ and, more specifically, dendrite-to-axon connections^9–11^. These technologies have been applied to induce single-neuron neurite differentiation^12^.

A typical method to control neuronal elongation is by microprinting patterns of extracellular matrix gel onto a nonadhesive surface in a cultivation dish. After creating predesigned photomasks with photolithography, casting a polydimethylsiloxane (PDMS) stamp, and inking cell adhesion molecules onto the stamp, the pattern is stamped onto a substrate, which is used to culture neurons, neurites and astroglial cells^1,3^. A quadrupolar microprinting pattern in a polylysine extracellular matrix gel was also used to guide the neurite differentiation of simple hippocampal neurons and showed the longest neurites became the axons^9^. Despite its simplicity, this method has limited flexibility. The microprinting pattern cannot be changed after the neurons have been seeded because the topology is defined by the predesigned photomask.

Recently, direct control of neurite elongation in predesigned patterns during cultivation, by using stamped patterns of thermoresponsive molecules and changing the temperature, and thus, the adhesivity of the pattern, was reported^13^. While these methods allow selective control over neuronal growth within the predesigned patterns, flexible control of cell-to-cell connections to form the desired networks was still not possible because of the limitations of microprinting technology. Another method, stepwise spot UV etching using a series of photoreactive layers on glass to control neurite growth of PC12 cell line cells, has been proposed; however, soluble extracellular matrix must be applied after each step of UV etching during cultivation within the predesigned patterns^14^.

Microstructural techniques use topographical confinement to control where neurons are positioned, which directions their neurites elongate, and overall pattern of the neuronal network. To form microstructures in the substrate, photolithography is used to create three-dimensional molds using an epoxy-based negative photoresist (such as SU-8) or PDMS. The direction of neuronal growth and connectivity is governed by the physical structure of the substrate^5,7^. This method has been used (1) to combine a semiconducting material with neurons to create a neuron semiconductor chip^4^, (2) to selectively control axonal elongation in asymmetric channels within a microfluidic chip^6^, and (3) to connect presynaptic and postsynaptic neurons through topologically heterogeneous PDMS architecture^15^. However, these technologies are also associated with the inability to alter the topological structure during cultivation, precluding directional control of dendrite-axon connections.

We have previously hypothesized that a photothermal microfabrication technique employing infrared lasers could be used to create microchambers, microchannels, and microtunnels by spot heating an agarose layer and have developed a photothermal microfabrication techniques that can be used to alter the structure of network patterns even during cell cultivation; thereby facilitating flexible changes in network patterns at a single-cell level^16^. In our previous studies, we used a focused infrared laser with a wavelength of 1480 nm, which is in the range of wavelengths that are absorbed by water, for direct etching of the agarose layer. However, the resolution of this method of spot heating the portion of the thin agarose layer within the light cone was limited by the wavelength-dependent diffraction limit of the infrared laser.

In addition, we have previously investigated using focused infrared laser with a wavelength of 1064 nm to form microtunnels at a thin chromium layer coated on the bottom surface of the agarose layer; only the portion of the agarose close to the bottom layer was melted because of the specific absorption properties. Using this photothermal microfabrication technology, we were able to successfully regulate the direction of individual axonal outgrowth and synaptic connections to form a network of rat hippocampal cells^17^. Multielectrode array measurement of external field potential in simple 16-cell lattice-shaped hippocampal networks constructed using photothermal microfabrication have demonstrated neighboring neurons with similar firing patterns^18^;post-tetanic facilitation was confirmed in straight-line eight-cell hippocampal networks, which gradually weakened and disappeared 24 h after stimulation^19^; and cardiomyocyte networks created in agarose photothermal microfabrication using human induced pluripotent-derived cells have been used as a quasi-in vivo assay to predict cardiotoxicity^20–22^.

Although these findings have demonstrated the flexibility of the technique for forming microstructures in specific positions on the chip during cell cultivation, the spatial resolution of agarose microfabrication remains limited and potential damage may be caused to cells close the focused laser’s heat because of its diffraction limit.

We hypothesize that an infrared laser with a wavelength of 1064 nm focused on an absorption target that is smaller than the wavelength of the irradiated infrared laser could be used to overcome these limitations. Because the spatial distribution of heat from the absorption of the spot heat source is independent of the wavelength and diffraction limit of light, the heated area will depend only on the shape of the absorption target, and temperature decays inversely proportional to the distance. The purpose of this study was to investigate and verify the parameters for a system with these characteristics.

## Results

### Photothermal microfabrication using a platinum-coated microneedle

**Figure 1:**
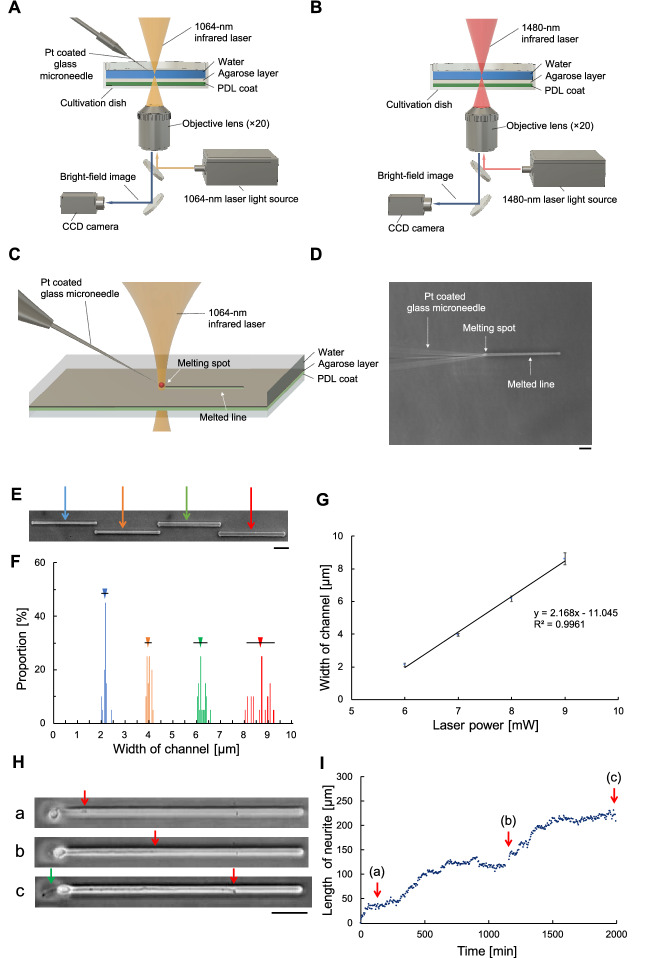
Photothermal microfabrication system: (**A**) Schematic of the new agarose microneedle etching system. (**B**) Schematic of a conventional agarose direct etching system. (**C**) In the new system, a platinum-coated microneedle is heated by the focused near-infrared laser to melt microchannels in the thin agarose layer. (**D**) A phase-contrast image showing spot heating from light absorption at the tip of the 0.7 μm-diameter microneedle . Bar,10 μm. (**E**) A micrograph of four 200 μm-long microchannels with 6 (blue arrow), 7 (orange arrow), 8 (green arrow) and 9 mW (red arrow) near-infrared lasers. Bar, 50 μm. (**F**) Microchannel widths—blue ([mean ± SD] 2.1 ± 0.1 μm), orange (4.0 ± 0.1 μm), green (6.1 ± 0.2 μm), and red (8.6 ± 0.4 μm) bars—correspond to each laser power—6 mW (blue), 7 mW(orange), 8 mW (green), and 9 mW, respectively. (**G**) The relationship between microchannel width and laser power. Error bars represent microchannel width SDs. (**H**) Elongation of a single neurite in the 2.2 μm-wide microchannel: (a) 2 h after cultivation (neurite length: 36 μm), (b) 20 h after cultivation (first neurite length 141 μm), and (c) 32 h after cultivation (first neurite length 231 μm. Red arrow and green arrow represent the leading edge of the first and second neurite, respectively. Bar, 50 μm. (**I**) Elongation time-course of the first neurite. The average elongation speed was 0.107 μm/min. Red arrows indicate 2, 20, and 32 h after cultivation.

The new microfabrication (agarose microneedle etching) system has a platinum-coated microneedle. A near-infrared laser with a wavelength of 1064 nm is focused by the microscope onto the tip of a 0.7 $\upmu \mathrm{m}$-diameter microneedle at the surface of the agarose layer (Fig. 1A). This wavelength (1064 nm) laser is not absorbed by water; therefore, heating occurs only at the tip of the microneedle. The agarose layer is melted by the heat, and the extracellular matrix (the bottom layer of the cultivation dish (poly-D-lysine)) is exposed. We compared the new microfabrication technique with a conventional direct etching microfabrication (agarose direct etching) system that uses an objective lens-focused 1480-nm infrared laser (Fig. 1B). Because the 1480-nm wavelength of the laser is absorbed by water, the agarose at the focus of is melted. In both systems, phase-contrast images of the agarose and the position of focused infrared laser are monitored with an infrared camera (charge-coupled device, CCD), and x-y location of the heated spot is controlled precisely using a computer-controlled motorized stage. The microneedle tip is positioned at the center of the focal point of the laser (Fig. 1C). Because diameter of the microneedle is less than the wavelength of the laser, the entire tip is located within the focal light cone; this is essential for stable heat conversion from light absorption. Both the position of microneedle and the focal point of the infrared laser were fixed; only the cultivation dish was shifted in the x-y plane with the motorized stage. The stage’s position sensors have a resolution of micrometer, and the timing of spot heating was controlled by the opening and closing of the laser’s shutter. For example, to form a microchannel, the cultivation dish was moved to a set start position. When the shutter opened a microchannel structure began to form underneath the microneedle’s tip because the agarose layer was melted. A homogeneous linear microchannel pattern was formed as the stage moved in the x-y plane. When the heat spot reached the predetermined endpoint, the shutter closed. Figure 1D is an example of a phase-contrast image during microchannel formation at a speed (20  $\upmu \mathrm{m}/\mathrm{s}$).

#### Microchannel width control

Microchannel width was controlled by changing the irradiation power of the infrared laser (Fig. 1(E)). The 200 $\upmu \mathrm{m}$ length microchannels formed by 6 mW, 7 mW, 8 mW, and 9 mW of power. Microchannel width (Fig. 1F) was proportional to the applied laser power (the slope of the fitted line in Fig. 1G was 2.17 $\upmu \mathrm{m}$/mW). Width distributions were calculated by measuring 20 points of each microchannel at 10 $\upmu \mathrm{m}$ intervals. When the 9 mW pattern was fabricated, the bottom surface of the plastic cultivation dish melted because of the high heat, and the width distribution was high; however, for 6 mW and 7 mW of power, width distributions were narrow, with standard deviations within0.1 $\upmu \mathrm{m}$. These results indicate that the width of microchannels can be easily and precisely control by modifying infrared laser power, as long as power is low enough to avoid damaging the cultivation dish.

#### Neurite elongation

Figure 1H shows an example of a single neurite in a 2.2 $\upmu \mathrm{m}$-wide microchannel. In our experiments, because the typical widths of neurites ranged from 1.9 to 2.3 $\upmu \mathrm{m}$, we used a width of 2.2 $\upmu \mathrm{m}$ for microchannels for single neurite elongation. Before the cultivation, we fabricated a round microchamber (20 $\upmu \mathrm{m}$ in diameter), and a microchannel (300 $\upmu \mathrm{m}$ by 2.2 $\upmu \mathrm{m}$) microchannel was added to the microchamber. Then, a single neuron was placed in microchamber. Two hours after cultivation started, the first neurite appeared and started to elongate into the microchannel at a speed of 0.11 $\upmu \mathrm{m}$/min (Fig. 1H(a)). Twenty hours after cultivation started, the length of first neurite was 36 $\upmu \mathrm{m}$ (Fig. 1H(b)). Thirty-two hours after cultivation started, the length of the first neurite was 231 $\upmu \mathrm{m}$ (Fig. 1H(c)). It should be noted that the second neurite was observed 16.6 h after cultivation started; however, it was remained in the microchamber and did not elongate. Figure 1I shows the elongation of a single neurite within the 2.2 $\upmu \mathrm{m}$-width microchannel, with a mean propagation velocity of 0.107 $\upmu \mathrm{m}$/min, which indicates the physical confinement of the 2.2 $\upmu \mathrm{m}$-width microchannel structure not seem to inhibit the elongation of single neurites.

### Spatial distribution of temperature

The temperature of the gel-state agarose must be raised to the melting point at the spot where the channel is to be formed. However, when temperature of a point rises, the temperature of the surrounding area also increases. For the non-destructive etching during cell cultivation, minimizing increases in temperature surrounding the heated spot is essential to avoid damaging cells.

In our conventional etching method, the heat from the 1480-nm focused infrared laser was absorbed by water. The agarose where the laser was focused melted. Because the wavelength-dependent diffraction limit of the condensing objective lens determined the intensity distribution of the laser, the spatial distribution of temperature was also widened by the diffraction limit. In the new system, because a wavelength of 1064 nm laser is essentially not absorbed by water, absorption heat occurs only on the tip of the microneedle at the focal point of the laser. A 0.7 $\upmu \mathrm{m}$-diameter microneedle was used because it is smaller than the 1064-nm wavelength of the laser, but for high-resolution spot melting, narrow spatial distribution of increased temperature is also essential. The surrounding temperature distribution of the microneedle tip heated by the 1064-nm wavelength laser should be narrower than that from direct spot heating of the agarose with a focused 1480-nm wavelength laser.

We compared the heat distribution of these two methods using the fluorescent quenching method with rhodamine B (a temperature-dependent fluorescent dye). Fluorescence of the dye decays as temperature rises, which is known as fluorescence quench^23^. Rhodamine B has been used for measurement of temperature distribution with micrometer resolution^24,25^.

The spatial distribution of fluorescence of a thin agarose layer covered with 2 mL of 100 $\upmu$ M rhodamine B and irradiated using an infrared laser with a wavelength of 1064 nm or 1480 nm was measured. The relative intensity($I_{R}$) of heat quenching was evaluated as:

1 $$\begin{aligned} \displaystyle I_{R}=\frac{I}{I_{RT}}, \end{aligned}$$

where *I* represents the measured rhodamine B intensity during infrared laser irradiation, and $I_{RT}$ represents measured light intensity at room temperature.

**Figure 2:**
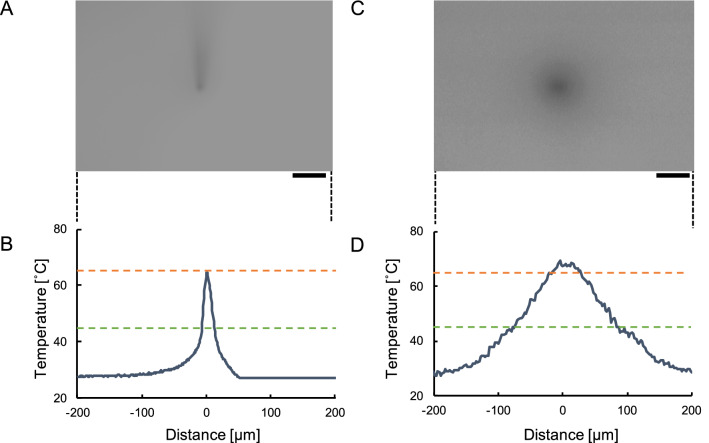
Spatial distribution of temperature around the spot heat source. (**A**) Fluorescence at the center of the image shows high temperature of the 0.7 $\upmu \mathrm{m}$-diameter platinum-coated glass microneedle that was the focal point of 9 mW of power from an infrared laser (wavelength: 1064 nm). Bar, 50 $\upmu \mathrm{m}$. (**B**) Temperature distribution corresponding to the x-axis of the image above, where x=0 represents the center of the microneedle. (**C**) Fluorescence at the center of the focal point of 35 mW of power from an infrared laser (wavelength: 1480 nm). Bar, 50 $\upmu \mathrm{m}$. (**D**) Temperature distribution corresponding to the x-axis of the image above, where x = 0 represents the center focal point of the infrared laser. Red dashed lines represent the melting temperature of agarose (65 $^\circ \hbox {C}$). Green dashed lines represent the temperature potentially damage neurons (45 $^\circ \hbox {C}$).

Figure 2A shows a fluorescent image of the platinum-coated 0.7 $\upmu \mathrm{m}$-diameter microneedle tip and its surroundings. The position of 65 $^\circ \hbox {C}$ in the intensity profile (Fig. 2B) was estimated at the interface between the melted agarose on the bottom surface of the cultivation dish because 65 $^\circ \hbox {C}$ is the melting point of agarose gel. The intensity of fluorescence at 45 $^\circ \hbox {C}$, which represents the range of temperatures that potentially damage cell, was estimated by interpolating the intensity of fluorescence at room temperature (27 $^\circ \hbox {C}$) and that at agarose melting point (65 $^\circ \hbox {C}$). The distance required for temperature to decrease from 65 $^\circ \hbox {C}$ to 45 $^\circ \hbox {C}$ was estimated to be 7.0 $\upmu \mathrm{m}$.

The temperature distribution of the infrared laser with a wavelength of 1480 nm (Fig. 2C) and its intensity profile (Fig. 2) was wider at the focal point than the intensity profile at the tip of the microneedle, because of the diffraction limit and the direct absorption of heat by the water. The distance required for temperature to decrease from 65 $^\circ \hbox {C}$ to 45 $^\circ \hbox {C}$ was 61.5 $\upmu \mathrm{m}$, which is an order of magnitude larger than that of microneedle tip absorption.

When the spot heat source is placed in water, the differential equation for the conduction of heat is

2 $$\begin{aligned} \displaystyle \vec {h}=\kappa \nabla T, \end{aligned}$$

where $\vec {h}$ is the flow of heat, $\kappa$ is the thermal conductivity, and *T* is the temperature. Under a steady heat-flow condition with heat source *s*, an equilibrium state of heat that is independent to time is produced:

3 $$\begin{aligned} \displaystyle \nabla \cdot \vec {h}=s. \end{aligned}$$

If we substitute Eq. (3) into Eq. (2), we have

4 $$\begin{aligned} \displaystyle \kappa \nabla ^2 T= s. \end{aligned}$$

As the spatial distribution of temperature (in x-axis) is

5 $$\begin{aligned} \displaystyle T(x) = \frac{s}{4\pi \kappa }\frac{1}{x}, \end{aligned}$$

the ratio of distances of two temperatures $x_{(T_1)}$ and $x_{(T_2)}$ depends only on the ratio of these two temperatures $T_1$ and $T_2$ in the environment $T_0$,

6 $$\begin{aligned} \displaystyle \frac{x_{(T_1)}}{x_{(T_2)}} = \frac{T_2-T_0}{T_1-T_0}. \end{aligned}$$

As shown in Fig. 2B, when the temperature of the water close to the heat source is maintained at 65 $^\circ \hbox {C}$ and the environment temperature is at 28 $^\circ \hbox {C}$, the width of 45 $^\circ \hbox {C}$ is only $(x_{{45}{\,^\circ \hbox {C}}}/x_{{65}{\,^\circ \hbox {C}}}=)$ 2.17 times larger than the distance of 65 $^\circ \hbox {C}$ from Eq. (6). Therefore, if the 65 $^\circ \hbox {C}$ is confined within the 2 $\upmu \mathrm{m}$ agarose microchannel width when the heat is produced by the microneedle, the distance to 45 $^\circ \hbox {C}$ from the center of the microneedle should be 2.17 $\upmu \mathrm{m}$, which is only 1.17 $\upmu \mathrm{m}$ wider than that of 65 $^\circ \hbox {C}$. It is independent of the wavelength of the laser and less than the wavelength of a 1480-nm laser. These results indicate another advantage of the microneedle etching method—, heat damage to the cells in the surrounding area is minimized regardless of the wavelength of the laser and its diffraction limit. This ratio of distances is independent of the thermal conductivity of the buffer medium as far as the absorbance is independent to the size and shape of objects.

As shown in Fig. 2D and explained above, the laser with a wavelength of 1480 nm showed a much wider distribution of heat caused by direct absorption and because of the diffraction limit, which is wavelength-dependent for both spatial resolution and heat distribution width.

We have indicated the tendency of proportional correlation between the applied laser power and the microchannel width in Eq. (5) can also explain the proportional correlation shown in Fig. 1G when *s* and $x_{{65}{\,^\circ \hbox {C}}}$ are applied to laser power and the microchannel width. The laser power and microchannel width represent the heat source supply and the width in which the temperature is greater than 65 $^\circ \hbox {C}$; Eq. (5) can satisfy this result.

In this experiment, we used fluorescence quenching to estimate the spatial distribution of heat with the assumption that the temperature is directly proportional to the intensity of fluorescence when the temperature is less than 70 $^\circ \hbox {C}$^25^. However, the difference between the experimental results and the theoretical estimation of temperature distribution might be caused by convection and diffusion of liquid water and fluorescent dye. Even with these influences, the results indicate that heat distribution from the microneedle is an order of magnitude narrower than that from the focused infrared laser.

### Formation of direction-controlled neuronal network patterns

In stepwise microfabrication to form neuronal networks, a sufficient distance between surrounding neurons and neurites and the heated spot must be conserved to prevent heat damage and maintain the survival ratio of neurons^17^. We improved the spatial resolution of agarose etching to 2 $\upmu \mathrm{m}$ in width, which is the size of single neurites. To confirm this improvement, we used the new method to guide a single neurite in a desired direction with stepwise spot melting of agarose during cultivation.

**Figure 3:**
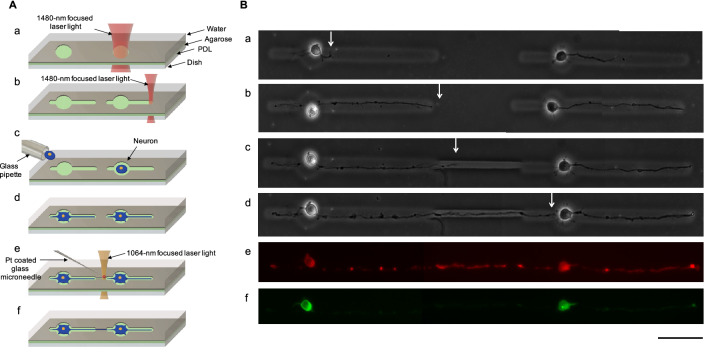
Direction controlled neurite elongation between two isolated single neurons. (**A**(a)) The Schematic drawing of 25 $\upmu \mathrm{m}$-diameter microchamber formation. (**A**(b)) Two closed-ended microchannels were formed for each microchamber. (**A**(c)) A single hippocampal cell was placed in each microchamber with a micropipette. (**A**(d)) A single neurite was elongated for each neuron. (**A**(e)) Microneedle etching was used to guide elongation of one neurite. (**A**(f)) The elongated neurite from one neuron connected to the other neuron. (**B**(a)) Phase-contrast image shows an E18 primary hippocampal neuron was placed in each microchamber (one day in vitro). (**B**(b)) Both first neurites in the left microchamber and right microchamber reached the right end of the microchannels after 3 days in vitro. (**B**)(c) Three hours after additional etching to connect the two closed-ended microchannels, the neurite from the neuron in the left microchamber entered into the new microchannel. (**B**(d)) The neurite reached the short neurite from the other neuron (4 days in vitro). Neurons (4 days in vitro) were fixed Tau-1 immunostained with Tau-1 to indicate axons (**B**(e)) and with MAP-2 to show cell bodies and dendrites (**B**(f)). White arrows indicate the leading edge of the neurite from the neuron on the left in the direction of neuron on the right. Bar, 50 $\upmu \mathrm{m}$.

A series of 25 $\upmu \mathrm{m}$-diameter microchambers with two closed-ended microchannels (50 $\upmu \mathrm{m}$ by 6.5 $\upmu \mathrm{m}$ and 150 $\upmu \mathrm{m}$ by 9.1 $\upmu \mathrm{m}$) were fabricated (Fig. 3A(a), A(b)). An E18 primary hippocampal neuron was placed in each microchamber (Fig. 3A(c)), and cultivation began (Fig. 3B(a)). After 3 days, neurites reached the end of the long microchannels (Fig. 3A(d), B(b)). An additional channel, with a width of 3.1 $\upmu \mathrm{m}$, was etched (using 7 mW of power) (Fig. 3A(e)). Three hours after, the neurite entered into the new microchannel (Fig. 3B(c)). On the next day, the neurite reached the other neuron (Fig. 3A(f), B(d)). To confirm differentiation of these neurites, we stained neurons with Tau-1 (for identifying axons) (Fig. 3B(e)) and MAP-2 (for identifying dendrites Fig. 3B(f)). Both long neurites (those extending to the right from each neuron had differentiated into axons (Fig. 3B(e)). The axon from the neuron on the left was successfully guided to the right neuron without causing damage to the left neuron from the heat of additional etching.

**Figure 4:**
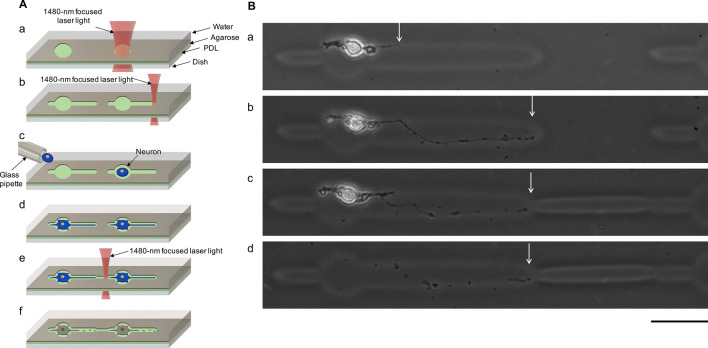
Damage caused by the focused 1480-nm infrared laser. (**A**(a)) Schematic drawing showing formation of 25 $\upmu \mathrm{m}$-diameter microchambers. (**A**(b)) Two closed-ended microchannel formation for each microchamber. (**A**(c)) A single hippocampal cell was placed in each microchamber. (**A**(d)) A single neurite was elongated for each neuron. (**A**(e)) Direct etching was used to guide elongated neurites. (**A**(f)) The neurite and neurons were damaged and were disappeared. (**B**(a)) Phase-contrast image shows a neurite elongated into the microchannel from a single neuron (2 days in vitro). (**B**(b)) The first neurite reached the right end of the closed-ended agarose microchannel (3 days in vitro). (**B**(c)) Three hours after additional direct etching, the cell body and neurite were still evident. (**B**(d)) The cell body and neurite disappeared (4 days in vitro). Each white arrow shows the position of the leading edge of the neurite. Bar, 50 ${\upmu \mathrm{m}}$.

As shown in Fig. 3B, apparent damage was not observed after additional etching, even though the neurite had reached the end of the closed-end of the microchannel just before additional microfabrication started. We also compared the heat damage to neurons during additional etching with the conventional direct 1480-nm focused infrared laser. The same patterns as those in Fig. 3 were prepared (Fig. 4A(a), A(b)). Similarly, an E18 primary hippocampal neuron was placed in each microchamber (Fig. 4(c)), and a neurite elongated in the right microchannel after 2days in vitro (Fig. 4B(a)). The first neurite elongated from the cell body reached the right end of the agarose pattern in 3 days in vitro (Fig. 4A(d) and B(b)). Three hours after additional etching with a 35-mW power 1480-nm wavelength infrared focused laser beam (Fig. 4A(e)), the cell body and the neurite were still observable 3 h after the additional microfabrication (Fig. 4B(c)); however, the cell body and neurite were not observed at 4 days in vitro (Fig. 4A(f) and B(d)). The results indicate that the heat from additional etching damaged the neurons and weakened adhesion. Thus, the influence of heat from the direct 1480-nm focused laser was more severe than that of the microneedle.

### Probabilistic control of first neurite elongation direction by width difference of microchannels

**Figure 5:**
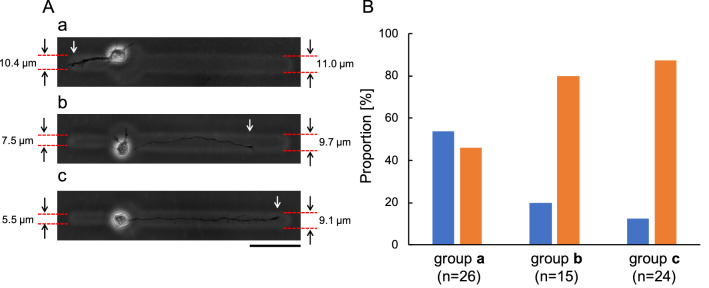
The relationship between the first neurite elongation direction and the widths of left and right microchannels. (**A**) The micrographs of single neuron cultivated for 36 h in each agarose microstructure with two closed-ended microchannels. The length of left microchannel from round-shaped microchamber was 50 $\upmu \mathrm{m}$, the length of right microchannel was 150 $\upmu \mathrm{m}$. (a) Group (a), two microchannels were approximately equal widths. The width of left channel was 10.4 $\upmu \mathrm{m}$, and the width of right channel was 11.0 $\upmu \mathrm{m}$. (b) Group (b), 2:3 width ratio. The width of the left channel was 7.5 $\upmu \mathrm{m}$, and the width of the right channel was 9.7 $\upmu \mathrm{m}$. (c) Group (c), 1:2 width ratio. The width of the left channel was 5.5 $\upmu \mathrm{m}$, and the width of the right channel was 9.1 $\upmu \mathrm{m}$. White arrows indicate the position of the leading edges of the elongated neurites. Bar, 50 $\upmu \mathrm{m}$. (**B**) Relationships between the difference in channel width and the direction of the first neurite elongation. Blue, left (narrow) microchannel; orange, right (wide) microchannel.

To control the first neurite elongation direction before additional fabrication, we have used probabilistic control to guide the first neurite by exploiting two different widths of microchannels connected to a single-cell cultivation microchamber (Fig. 5) .

Figure 5A(a)–A(c) are micrographs of single neurons in the microchambers with two closed-ended microchannels (36 h after cultivation started). The lengths of the left-side microchannels were 50 $\upmu \mathrm{m}$, and the lengths of the right-side microchannels were 150 $\upmu \mathrm{m}$. To confirm the effectiveness of the width difference, we prepared three types of microchannel pairs with different ratio widths (approximately equal, 3:4, and 1:2).

In Fig. 5A(a), an equal ratio (group **a**) was used. The width of left microchannel was 10.4 $\upmu \mathrm{m}$, and the width of right microchannel was 11.0 $\upmu \mathrm{m}$. The first elongated neurite entered into left microchannel. In Fig. 5A(b), a ratio of 3:4 (group **b**) was used. The width of the left channel was 7.5 $\upmu \mathrm{m}$, and the width of the right channel was 9.7 $\upmu \mathrm{m}$. The first elongated neurite entered into the wide microchannel. In Fig. 5c, we used a 1:2 ratio (group **c**). The width of the left microchannel was 5.5 $\upmu \mathrm{m}$, and the width of the right microchannel was 9.1 $\upmu \mathrm{m}$. The first elongated neurite entered into the wide microchannel. The results are summarized in Fig. 5B. In the group **a**, 53.8% of neurites elongated into the left microchannel, and 46.2% of neurites elongated into the right microchannel (n = 26) (Fig. 5B group **a**). In contrast, in group **b**, 20.0% of neurites elongated into the left narrow microchannel, and 80.0% of neurites elongated into the right wide microchannel (n = 15). Similarly, in group **c**, 12.5% of neurites elongated into the narrow microchannel, and 87.5% of neurites elongated into the wide microchannel (n = 24).

These results indicate that a 3:4 ratio between microchannel width can work effectively to guide the elongation direction of the first neurites. Hence, the similarity between the population ratio in group **b** and group **c** may also signify that the tendency of the first neurite to elongate in this manner was not only probabilistic manner but a sensitive selection mechanism of the first neurite in physically confined structures. In practice, we used this selectivity with respect to different width microchannels effectively in our experiments, and all the first neurites were guided in the desired direction.

## Discussion

In this report, we demonstrated an agarose microfabrication technique that exploits infrared light absorption of thin microneedle. We used an infrared laser with a wavelength of 1064 nm, which is not absorbed by water. Hence, absorption occurred only at the tip of the platinum-coated microneedle. We chose platinum to coat the microneedle because it is (1) easy to form a stable and homogeneous coat on the thin glass microneedle surface with spattering, (2) chemically stable and inactive, and it has (3) a sufficiently absorption rate of one micrometer infrared light. The diameter of microneedle was chosen to be 0.7 $\upmu \mathrm{m}$, which is smaller than the infrared laser’s wavelength (1064 nm), for two reasons: One, it is smaller than the applied infrared laser’s wavelength; therefore, the entire microneedle tip can be placed within the focal point infrared laser to give stable and homogeneous heat without strict position control. Two, it minimized the size of the spot heat source (two micrometer resolution, which is the typical single neurites’ widths).

Our previous reports introduced microfabrication technology for agarose with direct focusing of an infrared laser to fabricate microchannels during cultivation^17–19^; however, the heat of the focused infrared laser extended beyond the focal spot. The temperature rose higher than 45 $^\circ \hbox {C}$ in an area that extended more than ten times as wider as the spot’s size. Therefore, the neurites in the surrounding area were damaged, and apoptosis occurred (Fig. 4).

Microneedle etching has significant advantages to overcome the problems of direct, focused infrared laser etching. As shown in Fig. 2, the spatial distribution of the heated area is reduced. Hence, even when the neurites reached the end of the closed-end microchannel, additional etching could be used to guide neurites without inducing apoptosis. The spatial distribution was also improved by using a smaller spot heat source with at the microneedle tip. The minimum size of the focus area of the infrared laser is limited caused by the diffraction limit of optics. In contrast, the heat distribution of light absorption is dependent only on the size of the microneedle, and decays exponentially in space. Hence, in principle, this etching method is independent of the limitations of geometric optics, and only regulated by the heat conduction as shown in Eqs. (5) and (6).

From these findings, we also confirmed that local heat could be controlled by the heat absorption of light even though the absorption source size was smaller than the wavelength of the light source. The spatial distribution of heat caused by the light absorption of an object smaller than its wavelength showed reliable spatial attenuation, which prevents unnecessary heat damage to surrounding cells. We also have compared 0.7 ${\upmu \mathrm{m}}$-diameter tungsten microneedles and found that it demonstrates similar absorption and microfabrication abilities; however, its stiffness was lower than that of the glass microneedle, which made handling difficult.

Our results show the potential of this method for use in constructive neuronal network studies, which have until now, been accomplished partly with conventional microprinting or microfabrication technologies. This additional microfabrication method is simple and flexible, and can be combined with microprinted or microfabricated patterns in agarose without difficulty. It can serve the additional functions of time-course change of their spatial confinements.

We have also used this agarose coating and stepwise formation for multielectrode array chip measurements^19–22^. Because cells on the microelectrodes escape easily by their own spontaneous movement or collective cell migration on the flat plates, we formed agarose chambers surrounding the microelectrodes after coating the multielectrode array chip, and single cells were placed in each microchamber. The advantage of this etching method is that all processes proceed under the direct observation with microscopy. There is no need for complicated alignment procedures when this method is combined with other microprinting, micropatterning, or microelectrode-based analysis technologies for fully direction-controlled neuronal network studies.

In conclusion, we have examined the microneedle phtothermal etching technology for stepwise microfabrication of agarose layer for neurite elongation direction control during cultivation. When a 0.7 $\upmu \mathrm{m}$-diameter platinum-coated microneedle was used with 1064-nm focused laser absorption for additional stepwise network formation, we successfully guided neurite elongation with 2 $\upmu \mathrm{m}$ resolution and with no damage to neurons during agarose etching. This method can overcome and complement the potential limitations of conventional photothermal microfabrication.

## Methods

This study was conducted in strict accordance with the Act on Welfare and Management of Animals of the Ministry of the Environment, Japan. All animal experiments and protocols were approved by the Animal Experiment Committee of Waseda University (2019-A072 and 2020-A022,) and adhere ARRIVE 2.0 guidelines.

### Agarose photo-thermal etching microfabrication system

The new microneedle etching system consists of three parts: a phase-contrast microscope (IX-71 with $\times$20 phase-contrast objective lens, LCUPlanFL N, OLYMPUS, Tokyo, Japan) with a motorized x-y stage (BIOS-206T, SIGMA KOKI, Tokyo, Japan), a 1064-nm focused laser irradiation module (PYL-1-1064-M, IPG Laser, Burbach, Germany), and a microneedle fixed in the three-dimensional micromanipulator (MHW-3, NARISHIGE, Tokyo, Japan). The glass microneedles were drawn using a micropipette puller (P-2000, Sutter Instrument, Novato, CA, USA). Then, the tip of processed glass microneedle was coated with platinum using a fine autocoater (JFC-1600, JEOL, Tokyo, Japan). Two wavelengths of lights (520-nm visible light for phase-contrast microscopy and 1064-nm laser for photothermal etching) were used simultaneously. Phase-contrast images were acquired using a charge-coupled device CCD camera (CS230, OLYMPUS). The dichroic mirrors and lenses in the system were chosen for their suitability for these two wavelengths.

### Agarose microfabrication

The agarose microstructures in a 35 mm cultivation dish (AGC TECHNO GLASS Co., Ltd., Shizuoka, Japan) were prepared as follows. First, the dish was made hydrophilic with a plasma ion bombarder (PIB-20, Vacuum Device, Ibaraki, Japan). The dish was coated with poly-D-lysine (Sigma-Aldrich, St. Louis, MO, USA) diluted to 0.1 mg/mL with sterilized water, and 85 $\upmu \mathrm{L}$ of 2.5% agarose (BM-BIO BM Equipment, Tokyo, Japan) was spread spin-coated at 500 rpm for 3 s, then 3000 rpm for 18 s with spin coater (1H-D7, MIKASA, Tokyo, Japan) to form the 100 ${\upmu \mathrm{m}}$-thick agarose layer. Pure water (2 mL) was added to the agarose layer. A microneedle was placed and fixed with a micromanipulator, and the 1064-nm wavelength laser was focused onto the tip of the microneedle. The cultivation dish was moved by the automated stage at 0.02 mm/s.

### Heat distribution measurement

The heat distributions of each infrared laser were measured with rhodamine B (Sigma-Aldrich) solution on the agarose layer. A thin agarose layer was coated by 2 mL of 100 µM rhodamine B solution. Heat quenching was observed with a cooled charge-coupled-device camera imaging system (ORCA-ER, Hamamatsu Photonics, Shizuoka, Japan). The fluorescent filter was chosen to correspond to the wavelength of the laser.

### Cell cultivation

Rat hippocampal neurons were isolated and purified from 18-day-old Wister rat embryos (Tokyo Laboratory Animals Science, Tokyo, Japan) using Neuron Dissociation Solutions (FUJIFILM Wako Pure Chemical, Osaka, Japan). The hippocampal neurons were cultured within Neuron Culture Medium (FUJIFILM Wako Pure Chemical) in the agarose-patterned-poly-D-lysine coated dish at 37 $^\circ \hbox {C}$ in 5% $\mathrm{CO}_2$ at saturated humidity. The neurons were placed, one by one, into each round microchamber in the agarose with a fire-polished glass pipette.

### Cell observation

Neurons were observed using inverted optical microscopy (IX-71 with $\times$10 phase-contrast objective lens, UPLFLN10X2PH, OLYMPUS) equipped with a cooled charge-coupled-device camera imaging system (ORCA-ER, Hamamatsu Photonics).

### Immunofluorescence staining

The immunostaining procedure was conducted using a modified version of a method described in our previous research^17,19^. For immunostaining of axons, Anti-Tau-1 (Sigma-Aldrich) was used as the primary antibody and goat anti-mouse IgG2a cross-adsorbed secondary antibody, Alexa Fluor 555 (Thermo Fisher Scientific, Waltham, MA, USA) was used as the secondary antibody. For immunostaining of dendrites, Anti-MAP2 mouse monoclonal IgG1 (Sigma Aldrich) was used as the primary antibody and rabbit anti-goat IgG cross-adsorbed secondary antibody, Alexa Fluor 488 (Thermo Fisher Scientific) was also used as the secondary antibody. The fluorescent images were recorded with a cooled charge-coupled-device camera imaging system (ORCA-ER, Hamamatsu Photonics).
