# Publisher Correction: Stepwise neuronal network pattern formation in agarose gel during cultivation using non-destructive microneedle photothermal microfabrication

**DOI:** 10.1038/s41598-021-97263-x

**Published:** 2021-09-08

**Authors:** Yuhei Tanaka, Haruki Watanabe, Kenji Shimoda, Kazufumi Sakamoto, Yoshitsune Hondo, Mitsuru Sentoku, Rikuto Sekine, Takahito Kikuchi, Kenji Yasuda

**Affiliations:** 1 Department of Pure and Applied Physics, Graduate School of Advanced Science and Engineering, Waseda University, Tokyo, 169‑8555 Japan; 2 Department of Physics, School of Advanced Science and Engineering, Waseda University, Tokyo, 169‑8555 Japan

Correction to: *Scientific Reports* 10.1038/s41598-021-93988-x, published online 19 July 2021

The original version of this Article contained an error in Figure 1, where panel G did not display correctly. The original Figure 1 and accompanying legend appear below.

**Figure 1:**
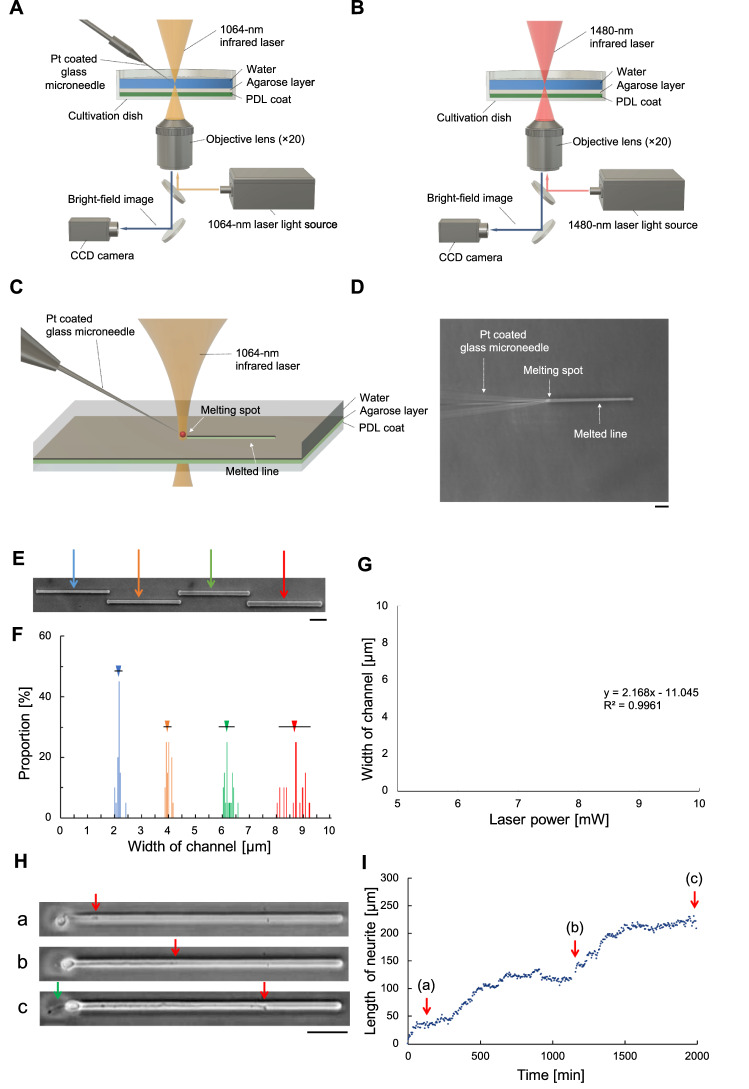
Photothermal microfabrication system: (**A**) Schematic of the new agarose microneedle etching system. (**B**) Schematic of a conventional agarose direct etching system. (**C**) In the new system, a platinum-coated microneedle is heated by the focused near-infrared laser to melt microchannels in the thin agarose layer. (**D**) A phase-contrast image showing spot heating from light absorption at the tip of the 0.7 μm-diameter microneedle. Bar, 10 μm. (**E**) A micrograph of four 200 μm-long microchannels with 6 (blue arrow), 7 (orange arrow), 8 (green arrow) and 9 mW (red arrow) near-infrared lasers. Bar, 50 μm. (**F**) Microchannel widths—blue ([mean ± SD] 2.1 ± 0.1 μm), orange (4.0 ± 0.1 μm), green (6.1 ± 0.2 μm), and red (8.6 ± 0.4 μm) bars—correspond to each laser power—6 mW (blue), 7 mW (orange), 8 mW (green), and 9 mW, respectively. (**G**) The relationship between microchannel width and laser power. Error bars represent microchannel width SDs. (**H**) Elongation of a single neurite in the 2.2 μm-wide microchannel: (a) 2 h after cultivation (neurite length: 36 μm), (b) 20 h after cultivation (first neurite length 141 μm), and (c) 32 h after cultivation (first neurite length 231 μm. Red arrow and green arrow represent the leading edge of the first and second neurite, respectively. Bar, 50 μm. (**I**) Elongation time-course of the first neurite. The average elongation speed was 0.107 μm/min. Red arrows indicate 2, 20, and 32 h after cultivation.

The original Article has been corrected.

